# Behavioural risk factors for non-communicable diseases among South African Durban-based refugees: a cross-sectional study

**DOI:** 10.1177/17579759231205852

**Published:** 2024-03-22

**Authors:** Jeanne Martin Grace, Mateisi Wailer Thabana

**Affiliations:** Discipline of Biokinetics, College of Health Sciences, Exercise and Leisure Sciences, University of KwaZulu-Natal, Durban, South Africa

**Keywords:** alcohol, behaviour change, diabetes, health care, nutrition, physical activity

## Abstract

Behavioural risk factors for non-communicable diseases (NCDs) are rising among refugees, increasing chronic disease prevalence that causes morbidity and mortality. This study aimed to ascertain the prevalence, awareness and management of behavioural risk factors for NCDs among South African Durban-based refugees. A once-off quantitative, cross-sectional design was conducted on the behavioural risk factors for NCDs among 122 randomly selected Durban-based refugees using a modified version of the World Health Organisation (WHO) STEPwise approach to NCDs surveillance (STEPS) instrument. Participants’ awareness and management of risk factors for NCDs were determined with a behavioural NCD awareness and management of behavioural NCD risk factor questionnaire. Smoking and alcohol prevalence were 4.1% and 20.7%, respectively, with 40.8% consuming fewer than five servings of fruit and/or vegetables daily. Participants performed more than 150 min of moderate physical activity per week. A significant 30.8% (*p* < 0.001) was aware that consuming alcohol poses an extremely large risk, similarly for smoking (38.7%; *p* < 0.001]. A significant 56.2% (*p* < 0.001) believe that regularly eating raw vegetables presents no risk, likewise for being physically active (51.7%; *p* < 0.001). A significant 40.6% (*p* < 0.001) of the participants always drink water or non-alcoholic drinks to manage their alcohol consumption, 54.2% (*p* < 0.001) manage their unhealthy diet by sometimes filling half their plates with fruits and vegetables, and 49.2% manage their physical activity levels by sometimes choosing a range of physical activities (*p* < 0.001). Refugees’ lack of awareness of behavioural risk factors for NCDs highlights the importance for health service providers to present health promotion programs to make refugees aware of their behavioural NCD’s risk factors and how it impacts their health.

## Introduction

The prevalence of non-communicable diseases (NCDs) and their risk factors is rising among refugees – a vulnerable and socially disadvantaged population – increasing their morbidity and mortality (1,2). A higher prevalence of chronic diseases among refugees has flowed into developing countries where NCD behavioural risk factors, such as tobacco use and harmful alcohol use, unhealthy diet and physical inactivity are associated with the development of diabetes, cardiovascular disease, cancers and chronic lung diseases (3). To control the global burden of NCDs, researchers suggest a shift to primary prevention by addressing inadequate nutrition, physical inactivity, alcohol consumption, smoking, high blood pressure and dyslipidaemia (4). The large influx of refugees in urban settings and the corresponding excessive burden of NCDs place significant pressure on under-resourced healthcare systems in low- and middle-income host countries like South Africa (5,6). Although South Africa does not have formal refugee camps, most refugees live in urban areas (7), such as Durban. Surprisingly, literature on South African-based refugees’ behavioural NCD risk factors and the awareness and management of their NCD risk factors are scant, which is concerning considering that NCDs have been determined as a significant health challenge among many humanitarian set-ups around the world (8).

Tobacco use is one of the main behavioural risk factors for NCDs and a leading cause of illness and death from major NCDs such as cancer, respiratory and cardiovascular diseases among Syrian refugees (9). The number of deaths is projected to grow to more than 8 million a year by 2030 if preventive measures are not implemented (10), with 86.9% of all deaths among current smokers in humanitarian settings (11). Researchers show that tobacco usage was 22.9% among Bangladeshi refugees aged 48.6 years (12), 31.4% among Syrian refugees aged 41.9 years (9), and 9.4% among Afghan refugees aged 36.8 years (13), notably lower than the high prevalence of 43.7% among the general population in Bangladesh (14). However, literature on the awareness and management of tobacco use among refugees is very scant.

Researchers showed that alcohol is the most widely used substance globally. In refugee camp settings, beer, wine and home-brewed alcohol are the most commonly consumed types of alcohol (15). In this regard, 98.6% of Syrian and 97.2% of Bangladeshi refugees indicated ever consuming alcohol (12,16). However, Durban-based refugees’ awareness and management of alcohol as an NCD risk factor are currently unknown.

Globally, the annual total number of deaths due to low fruit and vegetable consumption is 1.7 million (17,18). More than 95% of Syrian refugees (9) and 94.5% of Afghan refugees consume fewer than five servings of fruits and/or vegetables daily (13), in contrast to the much lower 76.3% of Bangladeshi refugees (12). To manage fruit and vegetable intake, it is recommended that a person should consume five servings of fruits and vegetables daily (19). Increased consumption of food high in salt and decreased consumption of vegetables and fruits can negatively impact the quality of refugees’ diets (9). A study by Rahman *et al*. (12) showed that 34.5% of Bangladeshi refugees consume extra salt in their food, and 37.2% of Syrian refugees add salt always/often to their meals before eating (16), which is similarly high, compared with a lower 28.1% of the Turkish general population who always add salt to their food before eating (20). To manage the risk of high salt intake, it is recommended that a daily intake of salt should be less than 5 g (19). Literature on the awareness and management of an unhealthy diet among refugees is sparse, with South African Durban-based refugees’ dietary habits currently yet to be discovered.

Physical inactivity contributes to 6% of deaths worldwide (21). It has become another health-related concern among refugees (22), contributing to an increase in the number of refugees being overweight and obese (21). Researchers evidenced that the prevalence of insufficient physical activity among Afghan refugees was 18.1% (13) and 8.1% among Syrian refugees (23). Studies on the physical activity levels among refugees are very scarce (1). However, 89.6% of Bangladeshi refugees’ physical activity levels are low (less than 150 min per week), and 6.8% and 2.4% perform moderate and vigorous physical activity, respectively (12). Although the physical activity levels of South African Durban-based refugees are unknown, minimum physical activity levels of either 150 min of moderate or 75 min of vigorous physical activity are recommended per week to obtain health benefits (24).

Studies focusing on behavioural NCD risk factors among South African-based refugees still need to be explored, with NCDs forming an integral part of health service provision even to this vulnerable population. Monitoring the current status of these key risk factors is an essential element of NCD control that could shed light on possible health promotion programs to be included in health service delivery. It is imperative to generate evidence to see how behavioural risk factors impact Durban-based refugees; hence, this study aimed to investigate the behavioural risk factors of NCDs amongst Durban-based refugees in South Africa.

## Materials and methods

### Study design and setting

A one-off, quantitative cross-sectional design survey was conducted among 122 Durban-based refugees living in the city of Durban. The study was conducted at the Pastoral Refugee Care Centre, Durban, Kwazulu-Natal, South Africa, as South African refugees are based mainly in urban areas and not restricted to refugee camps (7). Most adult African refugees migrate to South Africa for economic benefits and job opportunities, with around 75,512 refugees hosted, based on South Africa’s refugee statistics estimates (25). The majority in Durban originate from conflict-torn African countries like the Democratic Republic of Congo, Rwanda, Burundi, Angola, South Sudan, Ethiopia, Somalia and Zimbabwe. Unlike other African nations, South Africa lacks refugee camps, and many refugees live in urban areas around Durban, surviving without extensive assistance. Refugees have freedom of movement and can seek employment or self-employment, often starting informal sector enterprises due to limited opportunities in the formal sector.

The Refugee Pastoral Care Centre, affiliated with the Catholic Archdiocese of Durban, focuses on providing pastoral and social services to refugees, asylum seekers and migrants, aiming to restore their dignity, faith and hope through healing workshops and cohesion programs. The study site was chosen due to its location at the Denis Hurley Centre—a central hub for Durban’s large refugee population.

### Sampling

The study participants included refugees older than 18 years versed in English and living permanently in Durban, South Africa. Refugees meeting the specified criteria were included due to their English language proficiency, which is crucial for meaningful contributions to the study results. Refugees were excluded if their asylum was not secured or whose application was still in process, or who acquired citizenship through birth or marriage.

The sample size of 122 refugees was determined using a sample size calculator with parameters set at a 95% confidence level and a ±5% confidence interval (26). These participants were selected randomly to meet the minimum required sample size. To recruit participants, a sampling strategy was employed by contacting all organisations that assist refugees in attaining integration and independence. After obtaining informed consent, data were collected over 4 days at the Pastoral Refugee Care Centre.

### Data collection, measurements and process

#### WHO STEPwise approach to NCD risk factor surveillance instrument

Data were collected using a modified version of the WHO STEPwise approach to NCD surveillance (STEPS) instrument version 3.1 (28). STEPS was administered to the participants by the second author and trained research assistants who received adequate training in survey methodology. The instrument incorporated questions on behavioural and cardio-metabolic risk factors and is separated into four sections. The STEPS instrument’s first two steps were utilized for the current study’s purposes. Step 1 included questions on socio-demographic characteristics, and Step 2 explored the refugee’s behavioural risk factors such as tobacco use, alcohol consumption, physical inactivity and unhealthy diet (dietary habits).

##### Socio-demographic characteristics (WHO Step 1)

Socio-demographic characteristics, such as sex, age, the highest level of education, income, ethnicity and contact details, were collected first.

##### Self-reported behavioural risk factors (WHO Step 2)

Current use of tobacco (smoke and smokeless forms), alcohol consumption, and intake of fruits, vegetables and salt were collected subsequently. Physical activity at work, travel to and from places, recreational activities and time spent in sedentary behaviour were assessed using the Global Physical Activity Questionnaire (GPAQ) (27). Behavioural risk factors were determined based on the cut-offs recommended by STEPS guidelines (28). Tobacco (smoke or smokeless) and alcohol use in the last 30 days and 1 year were considered current use. Alcohol use was assessed as standard drinks (one standard drink = 100 ml wine, 285 ml beer or 30 ml Spirit/Toddy/Arrack).

#### Behavioural NCDs risk awareness questionnaire

Refugees’ awareness of their behavioural NCD risk factors (tobacco use, alcohol consumption, physical inactivity and unhealthy diet) was determined using a self-developed questionnaire. The questionnaire was piloted on 20 respondents not included in the study to ensure reliability and validity. For each risk factor, participants could select one of the following five options ‘No risk at all’, ‘A small risk’, ‘A medium risk’, ‘A fairly large risk’ and ‘An extremely large risk’.

#### Management of behavioural NCDs risk questionnaire

Refugees’ management of their behavioural NCD risk factors (tobacco use, alcohol consumption, physical inactivity and unhealthy diet) was determined using a self-developed questionnaire. The questionnaire was piloted on 20 respondents not included in the study to ensure reliability and validity. For each risk factor, participants could select one of the following five options ‘Never’, ‘Rarely’, ‘Sometimes’, ‘Often’, and ‘Always’ to indicate what they do to manage their behavioural risk factors.

#### Procedures

Field workers recruited from UKZN’s School of Health Sciences underwent training by a Biokineticist before data collection. The Durban Pastoral Refugee Care Centre organized a group meeting to share study aims and address concerns. Refugees received a thorough explanation of the study’s purpose and benefits before signing informed consent forms. A total of 122 refugees expressed interest by signing the consent forms.

Questionnaires were completed at the refugee centre during their first visit, and translated into English—a language spoken widely among the refugees. Data collected included awareness and management of NCD risk factors using the described tools.

### Statistical analysis

Data were analysed using the Statistical Package for the Social Sciences (SPSS, version 21.0, Chicago, IL). Descriptive statistics were presented as means and SD. Frequencies were represented in tables or graphs. A Chi-square goodness-of-fit (univariate) test was used on the categorical variables to test whether any response options were selected significantly more/less often than the others. Under the null hypothesis, it was assumed that all responses were selected equally. A binomial test was used to test whether a significant proportion of respondents selected one of a possible two responses. A one-sample *t*-test was used to test whether a mean score differed significantly from a scalar value. Statistical significance was set at *p* ⩽ 0.05.

### Results

A total of 121 refugees participated in the study, with a mean age of 40.5 (±8.6) years. The majority of participants, comprising 88 individuals (73.6%), were female. Additionally, 113 participants (93.3% lived in rural areas before being displaced from their origin. Supplemental Table 1 shows that the majority of participants, 93 (76.9%), earn less than R3800.00 per month; 58 (47.9%) of the respondents obtained a secondary school education. Most participants (81; 66.9%) originate from the Democratic Public of the Congo, with 79 (65.3%) unemployed; 63 (52.1%) of the participants are married.

### Behavioural NCD risk factors

#### Smoking and alcohol consumption

The majority of participants (116; 95.9%) do not smoke, and 117 (98.3%) do not smoke daily. Of the 122 participants, 96 (79.3%) do not consume alcohol, and only one participant (8%) consumes alcohol daily.

#### Unhealthy diet

In a typical week, fruit is eaten on an average of 3.87 (±1.89) days, while servings (number) of fruit on those days are 2.68 (±1.43). This follows that in a typical week, vegetables are eaten on an average of 4.39 (±1.90) days, with daily servings of vegetables being 3.02 (±1.86). Of the participants, 40.8% consume fewer than five servings of fruit and/or vegetables on average per day. Supplemental Table 2 shows that a significant 33.3% of the participants sometimes and 21.7% always add salt or a salty sauce to their food right before eating it. A significant 36.7% of the participants sometimes and 23.3% always add salt or a salty sauce when cooking or preparing food. Of the participants, a significant 35.8% never and 25% sometimes eat processed foods high in salt. Further analysis shows that the participants significantly perceive that they consume too little salt (M = 2.02, SD = 1.02, *t*(−10.5) = 118, *p* < 0.001).

#### Physical activity

A significant (66.9%) and (68. 1%) (*p* < 0.001) of the participants work does not involve continuous vigorous- and/or moderate-intensity activities at work during a typical week. A significant 67.7% and 71.3% (*p* < 0.001) of the participants, respectively, do not perform vigorous- and/or moderate-intensity sports, fitness or recreational (leisure) activities for at least 10 min.

During a typical week at work, the participants spent 4.51 (±1.88) and 4.53 (±2.05) days, respectively, on vigorous- and/or moderate-intensity activities as part of their weekly activities (Supplemental Table 3). The participants spent 209.18 (±271.49) and 252.95 (±254.06) min, respectively, performing vigorous- and/or moderate-intensity activities at work during a typical week. Supplemental Table 3 shows that, during a typical week, the participants partake respectively in vigorous- and moderate-intensity sports, fitness or recreational (leisure) activities for 3.18 (±2.08) and 3.45 (±2.11) days. The participants spent 117.58 (±110.78) and 121.55 (±115.42) min., respectively, performing vigorous- and/or moderate-intensity sports, fitness or recreational (leisure) activities during a typical day. Finally, during a typical day, the participants spend 129.65 (±173.97) min. sitting or reclining (being sedentary).

### Awareness of NCD risk factors

As portrayed in Table 1, a significant 56.2% (*p* < 0.001) of participants believe that regularly eating raw vegetables presents no risk at all. On the participant’s alcohol awareness, a significant 41.0%; (*p* < 0.001) believe that consuming alcohol poses no risk at all; however, 30.8% (p<0.001) believe it is an extremely large risk. A significant 51.7% (*p* < 0.001) of participants believe being physically active presents no risk at all. Regarding smoking, a significant 42% (*p* < 0.001) believe that smoking presents no risk at all, whereas 38.7% (*p* < 0.001) believe that it presents an extremely large risk.

**Table 1.** Awareness of behavioural non-communicable disease risk factors.

Behavioural item	Number of responses (%)					x^2^	df
	No risk at all	A small risk	A moderate risk	A fairly large risk	An extremely large risk		
Regularly eating fruit and vegetables	68 (56.2)*	13 (10.7)	24 (19.8)	4 (3.3)	12 (9.9)	107.47	4
Consumption of alcohol	48 (41.0)*	4 (3.4)	10 (8.5)	19 (16.2)	36 (30.8)*	57.23	4
Physical activity	62 (51.7)	22 (18.3)	21 (17.5)	9 (7.5)	6 (5.0)	83.58	4
Smoking	50 (42.0)*	2 (1.7)	6 (5.0)	15 (12.6)	46 (38.7)	86.08	4
Consuming foods that are high in salt	25 (20.8)	21 (17.5)	19 (15.8)	29 (24.2)	26 (21.7)	2.67	4

* *p* < 0.001.

### Management of behavioural NCD risk factors

Table 2 shows that, regarding the management of their tobacco use, a significant 76.7% (*p* < 0.001) of participants never avoid triggers; 78.6% (*p* < 0.001) never delay their tobacco craving by 10 min, 71.7% (*p* < 0.001) never chew on sugarless gum or hard candy, 64.4% (*p* < 0.001) never get physically active and 66.7% (*p* < 0.001) never do other things to manage their tobacco use.

**Table 2.** Management of behavioural non-communicable disease risk factors.

Behavioural item	Number of responses (%)					x^2^	df
Tobacco use	Never	Rarely	Sometimes	Often	Always		
Avoid triggers	33 (76.7)*	1 (2.3)	2 (4.7)	1 (2.3)	6 (14.0)	88.51	4
Delay your tobacco craving by 10 min	33 (78.6)*	0 (0)	2 (4.8)	2 (4.8)	5 (11.9)	64.87	3
Chew on sugarless gum or hard candy	33 (71.7)*	2 (4.3)	0 (0)	4 (8.7)	7 (15.2)	54.69	3
Get physically active	29 (64.4)*	1 (2.2)	4 (8.9)	3 (6.7)	8 (17.8)	58.44	4
Do other things to control your tobacco use	30 (66.7)*	0 (0)	3 (6.7)	1 (2.2)	11 (24.4)	46.64	3
Alcohol consumption	Never	Rarely	Sometimes	Often	Always		
Limit consumption of alcoholic drinks	29 (50.9)*	1 (1.8)	11 (19.3)	4 (7.0)	12 (21.1)*	41.51	4
Have a few alcohol-free days each week	33 (58.9)*	0 (0)	11 (19.6)	2 (3.6)	10 (17.9)	37.86	3
Drink water or non-alcoholic drinks	22 (34.4)*	1 (1.6)	8 (12.5)	7 (10.9)	26 (40.6)*	35.53	4
Keep non-alcoholic drinks at home	36 (57.1)*	1 (1.6)	4 (6.3)	5 (7.9)	17 (27.0)*	66.13	4
Do other things to control your alcohol consumption	31 (50.8)*	1 (1.6)	3 (4.9)	4 (6.6)	22 (36.1)*	59.57	4
Unhealthy diet (fruit and vegetables)	Never	Rarely	Sometimes	Often	Always		
Fill half your plate with fruits and vegetables	12 (10.0)	7 (5.8)	65 (54.2)*	8 (6.7)	28 (23.3)*	99.42	4
Cut back on solid fats and sugar	16 (13.3)	12 (10.0)	41 (34.2)*	22 (18.3)	29 (24.2)*	21.92	4
Get physically active	15 (12.5)	8 (6.7)	58 (48.3)*	12 (10.0)	27 (22.5)*	68.58	4
Do other things to control your diet	19 (16.4)	13 (11.2)	47 (40.5)*	15 (12.9)	22 (19.0)	32.62	4
Unhealthy diet (salt intake)	Never	Rarely	Sometimes	Often	Always		
Limit consumption of processed foods	20 (17.1)	7 (6.0)	52 (44.4)*	10 (8.5)	28 (23.9)*	55.52	4
Look at the salt content on food label	21 (18.3)	11 (9.6)	48 (41.7)*	11 (9.6)	24 (20.9)*	39.91	4
Buy low-salt alternatives	12 (10.3)	18 (15.5)	48 (41.4)*	20 (17.2)	18 (15.5)	34.69	4
Use spices other than salt when cooking	37 (31.1)*	16 (13.4)	37 (31.1)*	4 (3.4)	25 (21.0)*	33.73	4
Do other things to control your salt intake	28 (23.7)*	10 (8.5)	39 (33.1)*	12 (10.2)	29 (24.6)*	25.64	4
Physical activity	Never	Rarely	Sometimes	Often	Always		
Choose a range of physical activities	35 (29.2)*	12 (10.0)	59 (49.2)*	4 (3.3)	10 (8.3)	86.92	4
Set new fitness goals	43 (37.1)*	8 (6.9)	45 (38.8)*	10 (8.6)	10 (8.6)	62.36	4
Find a training partner or join a group activity	48 (40.7)*	5 (4.2)	47 (39.8)*	8 (6.8)	10 (8.5)	81.24	4
Walk a bit faster or choose a longer route	25 (21.0)*	10 (8.4)	51 (42.9)*	5 (4.2)	28 (23.5)*	54.74	4
Do other things to improve your physical activity	30 (25.6)*	8 (6.8)	47 (40.2)*	7 (6.0)	25 (21.4)*	47.40	4

* *p* < 0.001.

A significant 50.9% (*p* < 0.001) of participants never consume, and 21.1% (*p* < 0.001) limit the consumption of alcoholic drinks to manage their alcohol consumption (Table 2). A significant 58.9% (*p* < 0.001) of participants never have alcohol-free days, whereas a significant 34.4% (*p* < 0.001) of participants never or 40.6% (*p* < 0.001) always drink water or non-alcoholic drinks. Other participants manage their alcohol consumption by either never (57.1%, *p* < 0.001) or 27% (*p* < 0.001) always keep non-alcoholic drinks at home. Finally, a significant 50.8% (*p* < 0.001) of the participants never or 50.8% always (*p* < 0.001) do other things to control their alcohol consumption.

In managing their unhealthy diet on fruit and vegetable consumption, a significant 54.2% (*p* < 0.001) of participants sometimes or 23.3% always (*p* < 0.001) fill half of their plates with fruits and vegetables; 34.2% sometimes (*p* < 0.001) or 24.2% always (*p* < 0.001) cut back on solid fats and sugar; 48.3% sometimes (*p* < 0.001) or 22.5% always (*p* < 0.001) get physically active. Finally, a significant 40.5% (*p* < 0.001) sometimes do other things to manage their unhealthy fruit and vegetable consumption (Table 2).

Table 2 shows that regarding the management of their unhealthy diet (salt intake), a significant 44.4% (*p* < 0.001) of participants sometimes or 23.9% (*p* < 0.001) always limit the consumption of processed foods; 41.7% (*p* < 0.001) sometimes or 20.9% (*p* < 0.001) always look at the label’s salt content; whereas 41.4% (*p* < 0.001) sometimes buy low-salt alternatives, and a significant 31.1% (*p* < 0.001) never use spices other than salt when cooking. Finally, a significant 23.7% (*p* < 0.001) of participants never, 33.1% (*p* < 0.001) sometimes, and 24.6% (*p* < 0.001) always do other things specifically to manage their salt intake.

In managing their physical activity, a significant 29.2% (*p* < 0.001) of participants never and 49.2% (*p* < 0.001) sometimes choose a range of physical activities. A significant 37.1% (*p* < 0.001) of participants never or 38.8% (*p* < 0.001) sometimes set new fitness goals. Of the participants, 40.7% (*p* < 0.001) never or 39.8% (*p* < 0.001) sometimes find a training partner or join a group activity in managing their physical activity. A significant 21.0% (*p* < 0.001) of the participants never, 42.9% (*p* < 0.001) sometimes and 23.5% (*p* < 0.001) always walk a bit faster. Finally, a significant 25.6% (*p* < 0.001) of the participants never, 40.2% (*p* < 0.001) sometimes, and 21.4% (*p* < 0.001) always do other things to improve their physical activity.

### Discussion

This cross-sectional study described the behavioural risk factors for NCDs among South African Durban-based refugees and showed a low prevalence of behavioural risk factors for NCDs, with most refugees unaware that these risk factors pose a risk to their health. The findings indicated that 4.1% of the study population smoked, much lower than Bangladeshi and Syrian refugees (9,12). This low prevalence of smoking may result from 38.7% of the participants being aware that smoking poses ‘an extremely large risk’ and 12.6% indicating that it poses ‘a fairly large risk’. This is an interesting finding, as Jawad *et al*. (29) suggest that refugees smoke more than the general population. Our results are heartening when considering that other researchers found that 89.7% of the participants in their study were aware that smoking causes chronic obstructive pulmonary disease, 76.8% were aware that it causes stroke and 51% were aware that tobacco use increases the risk for type 2 diabetes (30).

Our study findings also showed that 79.3% of the participants do not consume alcohol, which is slightly lower compared with other researchers’ findings, showing that 98.6% of Syrian and 97.2% of Bangladeshi refugees indicated ever consuming alcohol (12,17). Our cohort’s lower alcohol consumption can be attributed to a moderately low 30.8% of refugees indicating that alcohol consumption poses ‘an extremely large risk’. The study’s results showed that 40.8% of our participants consume fewer than five daily servings of fruit and/or vegetables on a typical day compared with a high 94.5% of Bangladeshi refugees (12). It can be postulated that consuming at least five daily servings of fruits and vegetables, as recommended by the WHO, is a real challenge to the refugee population. Coinciding with the above postulation, 56.2% of the current study’s refugees indicated that not adhering to the WHO’s daily servings of fruits and vegetables intake recommendation poses ‘no risk at all’, showing their lack of awareness or knowledge of the WHO’s recommendations. A positive outcome of our study, considering that excessive salt intake is associated with hypertension, is that a lower proportion (21.7%) of participants reported always adding salt or salty sauce to their food just before eating, compared with the 37.2% reported among Syrian refugees (16). Similarly, a lower 23.3% always add salt or a salty sauce when cooking or preparing food compared with a higher 52.7% of Syrian refugees (16). The lower salt intake of our cohort can be ascribed to 41.4% of our study’s participants buying low-salt alternatives compared with the much lower 12.3% of Syrian refugees (16). The significance of sufficient daily fruit and vegetable intake and reduced salt consumption cannot be overstated. Researchers emphasise that increased salt intake and decreased fruit and vegetable consumption negatively affect refugees’ diets (9). Unfortunately, achieving this goal is challenging in South Africa due to expensive fruits and vegetables, exacerbated by inflation and a weakened currency.

The participants exceeded the recommendation of doing a minimum of 150 or 75 min of moderate or vigorous physical activity per week (24) by doing, respectively, 122 and 118 min of moderate- and vigorous-intensity sports, fitness or recreational (leisure) activities during a typical day. The much higher time spent doing moderate and vigorous-intensity physical activity can be attributed to the phrasing of the GPAQ question, which allowed participants to include their time spent performing recreational (leisure) activities. It can also be attributed to our cohort of refugees having less access to facilities like televisions, contributing to physical inactivity because of sitting and reclining when watching television. Corroborating our findings, researchers showed that 18% of Afghan refugees’ physical activity levels are low (13). One factor contributing to refugees’ having much higher physical activity levels than the general population is that circumstances force refugees to accept onerous and physically active jobs (13).

### Strengths and limitations

The study has strengths worth mentioning. It addressed a knowledge gap by quantitatively studying NCD risk factors among refugees in Durban, South Africa using a quantitative questionnaire, which allowed for capturing participants’ experiences compared with qualitative methods. Piloting ensured questionnaire appropriateness for refugees. However, limitations should be considered. Participant bias cannot be excluded, and causal relationships could not be determined due to the study’s quantitative nature. Self-reported data on sensitive issues like alcohol and smoking may be distorted. Generalisability is limited because participants represent only Durban-based refugees, not from all South African provinces. Additionally, two-thirds of respondents were female, potentially affecting the generalization of men’s health.

## Conclusions

The study’s findings indicate a low prevalence of behavioural risk factors for NCDs among South African Durban-based refugees. The results depict that this low risk is attributable to the various options the refugees execute to manage their behavioural NCDs risk factors. The latter finding is intriguing, showing a need for more awareness among most refugees who are not fully aware or believe that NCD risk factors pose a risk to their health. Many refugees were unfamiliar with the term NCD, but their understanding improved when presented with management options for NCD risk factors. The refugees’ lack of awareness of dietary habits and physical activities is concerning as it can negatively influence their management of NCD risk factors such as overweight and obesity, blood glucose, cholesterol and blood pressure. By implication, this population group’s lack of awareness regarding NCD risk factors renders them susceptible to NCDs. Raising awareness about NCD risk factors becomes essential for refugees to seek appropriate screening, management and prevention strategies. Therefore, implementing health promotion interventions to encourage healthy lifestyles and mitigate the adverse effects of NCD risk factors is crucial. This study’s expected contribution to research in this area will aid in improving management and preventive strategies for NCD risk factors among refugee populations. Current health service providers should consider including health promotion programs making refugees mindful of the impact of risk factors for NCDs.

## Supplemental Material

sj-docx-1-ped-10.1177_17579759231205852 – Supplemental material for Behavioural risk factors for non-communicable diseases among South African Durban-based refugees: a cross-sectional study Supplemental material, sj-docx-1-ped-10.1177_17579759231205852 for Behavioural risk factors for non-communicable diseases among South African Durban-based refugees: a cross-sectional study by Jeanne Martin Grace and Mateisi Wailer Thabana in Global Health Promotion

sj-docx-2-ped-10.1177_17579759231205852 – Supplemental material for Behavioural risk factors for non-communicable diseases among South African Durban-based refugees: a cross-sectional study Supplemental material, sj-docx-2-ped-10.1177_17579759231205852 for Behavioural risk factors for non-communicable diseases among South African Durban-based refugees: a cross-sectional study by Jeanne Martin Grace and Mateisi Wailer Thabana in Global Health Promotion

sj-docx-3-ped-10.1177_17579759231205852 – Supplemental material for Behavioural risk factors for non-communicable diseases among South African Durban-based refugees: a cross-sectional study Supplemental material, sj-docx-3-ped-10.1177_17579759231205852 for Behavioural risk factors for non-communicable diseases among South African Durban-based refugees: a cross-sectional study by Jeanne Martin Grace and Mateisi Wailer Thabana in Global Health Promotion
