# Supplementary material for: Behavioural risk factors for non-communicable diseases among South African Durban-based refugees: a cross-sectional study
Source: Glob Health Promot. 2024 Mar 22;31(3):90–100. doi: 10.1177/17579759231205852 (PMC11568683; doi:10.1177/17579759231205852)
Supplement: sj-docx-1-ped-10.1177_17579759231205852 – Supplemental material for Behavioural risk factors for non-communicable diseases among South African Durban-based refugees: a cross-sectional study [file sj-docx-1-ped-10.1177_17579759231205852.docx]

**Table 1. Socio-demographic characteristics**

| **Variable** | **n** | **Percentage (%)** |
| --- | --- | --- |
| **Income level per month**  <R3800  R3800 - R5000  R5 001 - R10 000  R10 001 - R15 000  >R25 000 | 93  12  4  2  2 | 76.9  9.9  3.3  1.7  1.7 |
| **Level of education**  Some/all primary school  Some/all secondary school  Certificate  Undergraduate degree/diploma  Postgraduate degree/diploma | 13  58  15  19  14 | 10.7  47.9  12.4  15.7  11.6 |
| **Country of origin**  Burundi  Congo  Democratic Republic of the Congo  Liberia  Mozambique  Rwanda | 22  12  81  1  1  1 | 18.2  9.9  66.9  8.0  8.0  8.0 |
| **Employment status**  Unemployed  Self-employed  Full-time employed  Part-time employed | 79  31  3  5 | 65.3  25.6  2.5  4.1 |
| **Marital status**  Never married  Married  Separated/Divorced  Widowed  Co-habiting | 38  63  12  6  2 | 31.4  52.1  9.9  5.0  1.7 |
