# Supplementary material for: Behavioural risk factors for non-communicable diseases among South African Durban-based refugees: a cross-sectional study
Source: Glob Health Promot. 2024 Mar 22;31(3):90–100. doi: 10.1177/17579759231205852 (PMC11568683; doi:10.1177/17579759231205852)
Supplement: sj-docx-2-ped-10.1177_17579759231205852 – Supplemental material for Behavioural risk factors for non-communicable diseases among South African Durban-based refugees: a cross-sectional study [file sj-docx-2-ped-10.1177_17579759231205852.docx]

**Table 2. Unhealthy diet (salt intake) as a behavioural non-communicable disease risk factor**

| **Behavioural item**  Number of responses and % x^2^  df | | | | | | | | |
| --- | --- | --- | --- | --- | --- | --- | --- | --- |
| **Unhealthy diet**  **(Salt intake)** | Never | Rarely | Sometimes | Often | Always |  |  |  |
| How often do you add salt or a salty sauce to your food before eating it? | 23 (19.2) | 22 (18.3) | 40 (33.3)* | 9  (7.5) | 26 (21.7)* | 20.42 | 4 |  |
| How often is salt or a salty sauce added to your household's cooking or preparing foods? | 14 (11.7) | 19 (15.8) | 44 (36.7)* | 15 (12.5) | 28 (23.3)* | 25.92 | 4 |  |
| How often do you eat processed food high in salt? | 43 (35.8)* | 22 (18.3) | 30 (25.0)* | 12 (10.0) | 13  (10.8) | 27.75 | 4 |  |

* p<.001
