# Supplementary material for: Behavioural risk factors for non-communicable diseases among South African Durban-based refugees: a cross-sectional study
Source: Glob Health Promot. 2024 Mar 22;31(3):90–100. doi: 10.1177/17579759231205852 (PMC11568683; doi:10.1177/17579759231205852)
Supplement: sj-docx-3-ped-10.1177_17579759231205852 – Supplemental material for Behavioural risk factors for non-communicable diseases among South African Durban-based refugees: a cross-sectional study [file sj-docx-3-ped-10.1177_17579759231205852.docx]

**Table 3. Physical activity as a behavioural non-communicable disease risk factor (Time and days spend performing moderate and vigorous physical activity at work, doing sports, fitness, recreational activities and being sedentary)**

| **Time and days spent performing moderate and vigorous physical activity at work (days/minutes)** | **n** | **Min/Max** | **Mean/**  **SD** | **95% CI** |
| --- | --- | --- | --- | --- |
| In a typical week, how many days do you do vigorous-intensity activities at work (minutes)? | 35 | 1/7 | 4.51  (1.88) | 3.86-  5.16 |
| How much time do you spend doing vigorous-intensity activities at work (minutes)? | 39 | 0/1440 | 209.18 (271.49) | 121.17-  297.19 |
| In a typical week, how many days do you do moderate-intensity activities at work? | 32 | 1/8 | 4.53  (2.05) | 3.79-5.27 |
| How much time do you spend doing moderate-intensity activities at work (minutes)? | 37 | 0/960 | 252.95 (254.06) | 168.24-  337.66 |
| **Time and days spend performing moderate and vigorous physical activity performing sports, fitness and recreational activities (days/minutes)** | **n** | **Min/Max** | **Mean/**  **SD** | **95%**  **CI** |
| How many days do you do vigorous-intensity sports, fitness or recreational (leisure) activities in a typical week? | 34 | 1/7 | 3.18  (2.08) | 2.45-  3.91 |
| How much time do you spend doing vigorous-intensity sports, fitness or recreational activities on a typical day (minutes)? | 38 | 0/420 | 117.58 (110.78) | 81.17-  153.99 |
| In a typical week, how many days do you do moderate-intensity sports, fitness or recreational (leisure) activities? | 31 | 1/7 | 3.45  (2.11) | 2.68-4.22 |
| How much time do you spend doing moderate-intensity sports, fitness or recreational activities on a typical day (minutes)? | 33 | 0/420 | 121.55 (115.42) | 80.62-  162.48 |
| How much time do you usually spend sitting or reclining on a typical day (minutes)? | 121 | 0/765 | 129.65 (173.97) | 98.34-  160.96 |
